# A geophysics-informed pro-poor approach to earthquake risk management

**DOI:** 10.1007/s11069-024-06983-6

**Published:** 2024-12-16

**Authors:** Himanshu Agrawal, Chenbo Wang, Gemma Cremen, John McCloskey

**Affiliations:** 1 School of Geosciences, University of Edinburgh, Edinburgh, UK; 2 Department of Civil, Environmental and Geomatic Engineering, University College London, London, UK

**Keywords:** Earthquake risk management, Pro-poor, Decision support environment, Geophysics-informed policies

## Abstract

Recent earthquake disasters have highlighted an urgent need for continuous advancements in approaches to reducing seismic risk. Decision-making on such strategies should consider subsurface geophysical information (e.g., seismic site response), given its direct link to seismic hazard. This may be particularly important in regions where the poorest in society often reside in areas with softer soils that lead to higher ground-motion amplifications. In this context, we propose a framework to support decision-making on earthquake risk policies, which explicitly integrates information on the geophysics of an urban system as well as its physical and social environment. The framework is based on the Tomorrow’s Cities Decision Support Environment, which was designed to support urban planning with a focus on pro-poor disaster risk reduction in countries of the Global South. It is further underpinned by a cost–benefit analysis, which facilitates the assessment of potential policies in terms of both their ability to reduce earthquake risk as well as their value for (often limited) money. We illustrate the framework using a well-established virtual urban testbed based on Global South cities, which reveals that geophysics-informed policy making can successfully lead to pro-poor earthquake risk reduction.

## Introduction

Earthquake disasters have far-reaching consequences that include extensive infrastructure damage, economic losses, and various other hardships (Allen et al. 2009; CRED-UNDRR 2020). Recent examples of such disasters are: (1) the 2023 Western Nepal Earthquake (UNRCO 2023), a relatively moderate seismic event (with moment magnitude ${M}_{w} 6.4$) that nevertheless resulted in approximately 150 deaths, around 27,000 completely damaged residential buildings, and 250,000 people requiring humanitarian assistance; (2) the 2023 Turkey-Syria earthquakes (maximum ${M}_{w}$ 7.8), resulting in more than 44,000 deaths, over 100,000 injuries and approximately 160,000 building collapses (Miryam 2023; Yu et al. 2024); and (3) the 2023 Morocco earthquake (${M}_{w}$ 6.8), leading to approximately 3,000 deaths and hundreds injured or missing (Cheloni et al. 2024). Growing populations, rapid urbanisation, and amplifying physical vulnerability (caused by unplanned/informal modifications to the urban layout and ageing infrastructure) mean that the risk of earthquake disasters is constantly increasing worldwide (Feldmeyer et al. 2017; Pesaresi et al. 2017). All of these emphasise an urgent need to better protect urban systems against the challenges caused by earthquake events (Du et al. 2023; Freddi et al. 2021).

To address this requirement, policymakers can implement various risk reduction strategies, categorised as either ‘soft’, such as early warning, disaster financing, insurance, or ‘hard’, such as structural retrofitting (Freddi et al. 2021; Kiyono 2021; Mesta et al. 2023; Zhang et al. 2022). The effectiveness of any such policies can be evaluated through quantitative earthquake risk assessments, which involve models that link geophysical (earthquake hazard) data with engineering and socioeconomic information to produce various (probabilistic) impact measurements for potential seismic events (Baker et al. 2021). The accuracy of these assessments is continuously enhanced through lessons learned from previous earthquakes (Atmaca et al. 2020; Meguro 2015; Patwary et al. 2023; Reyners 2011; Zhang et al. 2022). Their advancement is further supported by improving computational capabilities, which facilitate the development of sophisticated, high-fidelity risk models that can handle a wide range of parameters and simulations (McCloskey et al. 2023). However, state-of-the-art risk assessment approaches still encounter several shortcomings related to modelling, data and underlying assumptions (Galasso et al. 2021). One of the most critical limitations is their frequent inability to account for the disproportionate impacts of earthquake disasters on the most vulnerable in society (Baker 2012; Hallegatte et al. 2020; Soden et al. 2023; Loos et al., 2023; Walsh and Hallegatte 2020; Markhvida et al., 2020). This is a particularly serious shortcoming in the context of Global South countries, where socio-economic inequalities are highly pronounced (Dodman et al. 2012; Hasell et al. 2023) and the poorest in society lack basic resources (food, medicine, standby generators, etc.) for disaster preparedness (Levac et al. 2012). Consequently, there is an increasing emphasis on the need to integrate pro-poor thinking and approaches in disaster risk management (DRM) and assessment (Unger et al. 2017; Wehmer 2012), which is also recognised in the guiding principles of the 2015–2030 Sendai Framework for Disaster Risk Reduction (UNISDR, 2015). Examples of practical pro-poor DRM strategies include cash grant schemes, microfinance programs, low-interest loans, subsidised or flat-rated catastrophe insurance, home retrofitting/reconstruction, and managed relocation programs that specifically target low-income populations (Parvin and Shaw 2013; Tiwari 2015; Matias et al. 2018; Du and Greiving 2020; Quigley et al. 2020; van Es and Bruins 2023; Valle, 2024).

Earthquake risk reduction strategies often take advantage of geophysical information directly related to seismicity, such as site response with respect to ground shaking intensity, near-field conditions, and susceptibility to ground instability (e.g., liquefaction and landslide). For example, seismic design building codes typically account for local site conditions through different site classes, which are associated with different design spectra (Verdugo 2019). At a regional scale, geophysical information has been leveraged to support decision-making related to risk-sensitive urban planning, building retrofitting programs, earthquake-related emergency response planning, and preemptive community relocation across various regions with high seismic hazard (Ansal and Tonuk 2006; Barua et al. 2023; Celikbilek and Sapmaz 2016; Dolce et al. 2021). However, the utility of geophysical information in earthquake DRM remains generally unclear amongst a myriad of other relevant (e.g., engineering, economic, planning) data (Quigley et al. 2020). This is a particularly critical gap in understanding for regions where the urban poor are often pushed by land scarcity and/or market dynamics to reside in risky locations such as low-lying soft-soil floodplains close to rivers (Nakagawa et al. 2009; Boelhouwer et al., 2018; Shi and Naylor, 2023; Bangalore et al. 2019; Kawasaki et al. 2020), which in turn have high seismic amplification characteristics.

This study addresses the aforementioned shortcomings of conventional seismic risk assessment and related DRM, providing a framework for investigating how geophysics-informed policymaking may affect the seismic risk of different socio-economic groups (compared to spatially independent or more explicit income-based risk-mitigation interventions). The framework is adapted from that of Wang et al., (2023) – which focuses on the design of risk-sensitive, pro-poor policies and is based on the Tomorrow’s Cities Decision Support Environment (TCDSE; Galasso et al. 2021; Cremen et al. 2023)-to: (1) explicitly integrate geophysical information; and also (2) consider the efficiency of policies in reducing earthquake impact on different populations (through a cost–benefit analysis), recognising the importance of funding constraints in real-life decision-making processes (Kenny 2012). We use the framework to assess policies in the earthquake-prone virtual urban testbed “Tomorrowville”, which imitates a Global South urban setting through its physical and socio-economic characteristics (Menteşe et al. 2023).

The remainder of the paper is organised as follows. In Sect. 2, we present the proposed framework, specifically highlighting the novel components introduced in this study. Section 3 details an application of the framework to a well-established virtual urban testbed. Finally, Sect. 4 concludes with a discussion of the insights gained from the case study.

## Framework

**Fig. 1:**
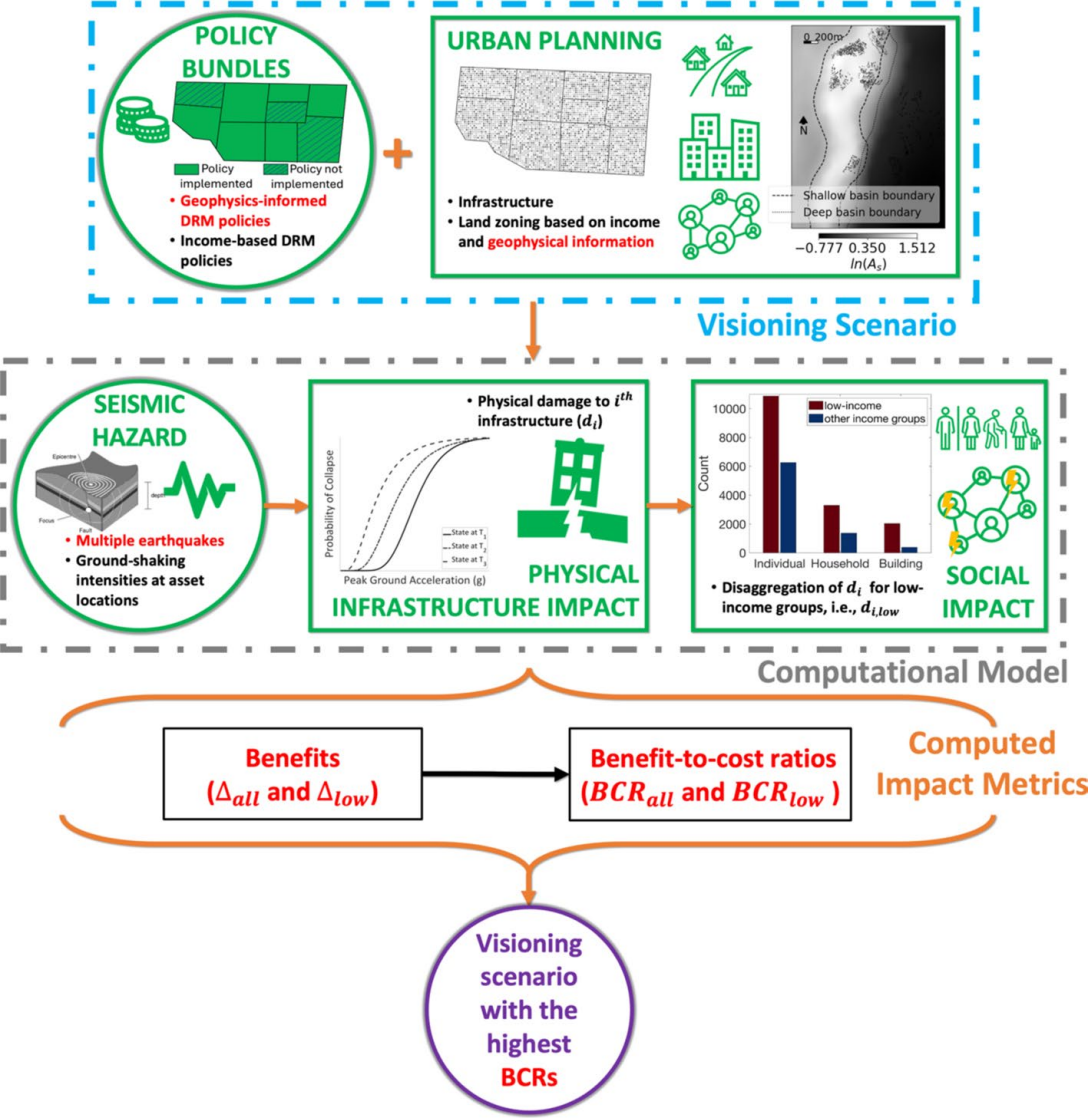
The proposed framework for assessing geophysics-informed policy making in terms of reducing seismic risk across different socio-economic groupings, which is adapted from Wang et al. (2023). Novelties (specific modifications to the framework proposed by Wang et al. 2023) are highlighted in red

The proposed framework (see Fig. 1) is based on that introduced by Wang et al., (2023), which investigates the pro-poorness of earthquake risk-sensitive policies in a (potentially future) urban context of interest and offers a general simulation-based framework for earthquake-related soft policy design. The Wang et al., (2023) framework contains a series of data and calculation modules/stages that are substantially modified/enriched in this study to specifically assess geophysics-informed and income-based (as well as more spatially independent) policies in terms of reducing seismic risk across various socio-economic groupings.

The *Urban Planning* module incorporates detailed, georeferenced information on the physical, environmental and social features underpinning the urban context (at a prescribed temporal instance). A *Visioning Scenario* is then generated by reflecting the implementation of a geophysics-informed, income-based or spatially independent policy (bundle) in the detailed urban plan. Seismic hazard calculations are conducted in the *Seismic Hazard Modelling* module to determine the physical (*Physical Infrastructure Impact* module) and social (*Social Impact* module) impacts of earthquakes on the *Visioning Scenario*. The *Computed Impact Metrics* stage assesses: (1) the effectiveness of the policy (bundle) in terms of the seismic impact reduction that results from its implementation across all (${\Delta }_{all}$) and low-income (${\Delta }_{low})$ populations; and (2) the efficiency of the policy (bundle), using benefit–cost ratio metrics (${BCR}_{all}$ and ${BCR}_{low}$) that account for both its effectiveness and financial implications. A series of different *Policy Bundle* policy configurations (and therefore *Visioning Scenarios*) may be assessed; the best *Visioning Scenario* is then deemed to be the one with the highest efficiency. We now provide further discussion of the framework modules/stages, emphasising their specific interpretation in the context of this study. This means that the descriptions of the proposed framework’s modules are not exhaustive; information contained in existing papers on the TCDSE – Cremen et al. (2023), Gentile et al. (2022a), Mentese et al. (2023), and Wang et al., (2023)—will not be repeated for brevity.

### Urban planning

In addition to relevant earthquake exposure data that is described in Wang et al. (2023) and Cremen et al. (2023), this module also includes pertinent geophysical information that represents the ground motion response of the soil and its amplification characteristics. Examples of such information include the parameters of a sub-surface seismic velocity model and associated seismic site response that can be obtained through seismic microzonation studies (Sitharam and Anbazhagan 2008; Verdugo 2019), which may be represented through summary metrics (e.g., time-averaged shear-wave velocity in the upper 30 m; ${V}_{s30}$, Chung and Rogers, 2012).

### Policy bundles

The *Policy Bundles* module contains various geophysics-informed and/or income-based (as well as spatially independent) policies applied to the conditional urban plan of the *Urban Planning* module. The policies are developed and applied based on physical, socio-economic and/or geophysical information stored in the *Urban Planning* module. These policies can be implemented either separately or together as a bundle. In addition to prospective and compensatory policies discussed and/or assessed in previous studies (Cremen et al. 2023 and Wang et al. 2023), these policies could be corrective in nature, i.e., reducing existing risk, for example, through retrofitting existing buildings or facilitating mass relocation of vulnerable communities at risk. The overall purpose of the framework centres on assessing the policies included in this module; various policy bundles (each containing one or more policies) can be examined, producing distinct *Visioning Scenarios*.

### Seismic hazard modelling

The *Seismic Hazard Modelling* module uses the geophysical information of the *Urban Planning* module in conjunction with relevant seismological data (e.g., earthquake-rupture parameters) to produce spatial ground-shaking hazard maps (ground-motion fields) in terms of intensity measures (IMs) such as Peak Ground Acceleration (PGA). The specific IMs required depend on the fragility models used to characterise physical impacts (see Gentile et al. 2022a, for a comprehensive list of IMs that may be necessary to compute).

### Physical infrastructure impact

The *Physical Infrastructure Impact* module synthesises damage for the *i*th piece of infrastructure into an appropriate scalar (possibly normalised) measure, ${d}_{i}$, which represents the output of this module. If the seismic hazard is scenario-based, ${d}_{i}$ could (for example) represent the corresponding average damage state, i.e., for a single (deterministic) ground-motion field *im:*

1 $$\begin{array}{c}{d}_{i}=\sum_{j=1}^{{n}_{ds}}d{s}_{j}\times p(d{s}_{j}|im)\end{array}$$

where $d{s}_{j}$ is the *j*th damage state, ${n}_{ds}$ is the number of considered damage states, and $p(d{s}_{j}|im)$ is the conditional probability of $d{s}_{j}$ occurring that is derived from the fragility relationships. If the seismic hazard is time-based, ${d}_{i}$ could instead represent the expected annual damage state, for instance. Note that ${d}_{i}$ can relate to any type of engineered asset and can therefore be used to capture potentially cascading impacts across multiple critical pieces of critical infrastructure (Alexander and Pescaroli 2019). ${d}_{i}$ should be appropriately normalised if infrastructure with different numbers of damage states is collectively considered.

### Social impact

The *Social Impact* module uses socio-economic data and information on individuals’ interaction within the built environment from the *Urban Planning* module, to distinguish the set of ${d}_{i}$ associated with infrastructure exclusively linked to low-income populations (denoted as ${d}_{i,low}$) from the complete distribution of ${d}_{i}$.

### Computed impact metrics

The *Computed Impact Metrics* module uses ${d}_{i}$ and ${d}_{i,low}$ to assess the effectiveness and efficiency of the policies introduced in the *Policy Bundle*. Effectiveness is defined as the reduction in earthquake impact (represented through ${d}_{i}$ and ${d}_{i,low}$) associated with the *Policy Bundle* policies (*p*), relative to the case in which these policies are absent (*o*). It is defined using two metrics ${\Delta }_{all}$ and ${\Delta }_{low}$, where:

2 $$\begin{array}{c}{\Delta }_{x}={\int }_{0}^{\text{max}({d}_{x})}(F{\left({d}_{x}\right)}_{p}- F{\left({d}_{x}\right)}_{o})\,{ \text{d}d}_{x}\end{array}$$

**Fig. 2:**
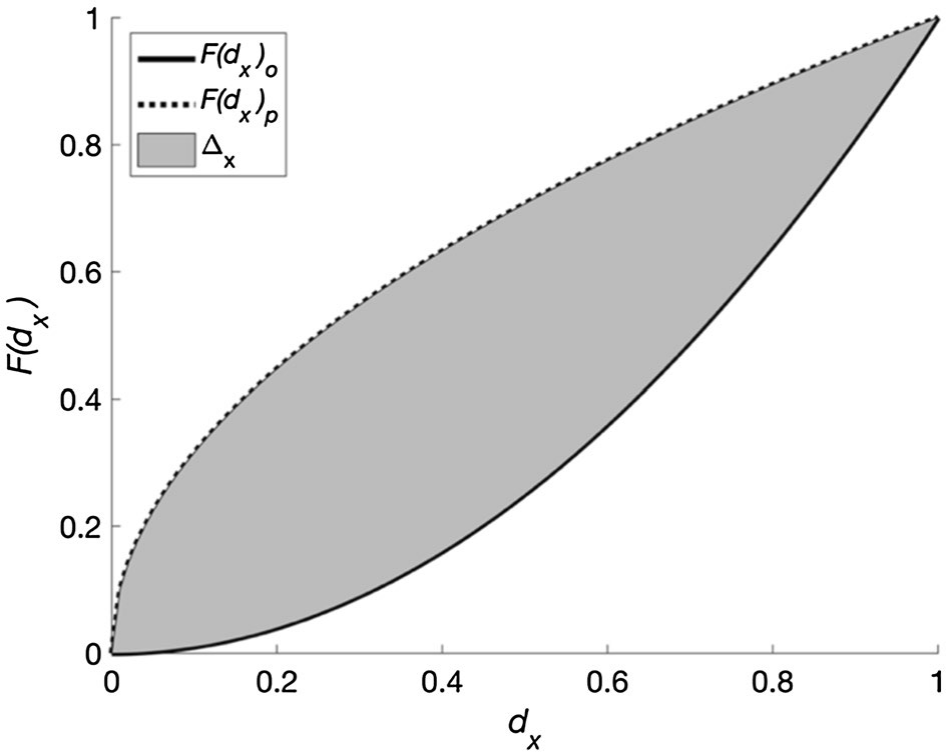
Example sketch of ${\varDelta }_{x}$ (denoted with hatched marking), which is the reduction in the area above the empirical CDF of ${d}_{x}$- $F{\left({d}_{x}\right)}_{o}$- that results from policy intervention *p*. All variables are defined in the text

$x$ represents “all” or “low”, ${d}_{x}={d}_{i}$ for ${\Delta }_{all}$, and ${d}_{x}={d}_{i,low}$ for ${\Delta }_{low}$. $F{\left({d}_{x}\right)}_{o}$ is the empirical cumulative distribution function of ${d}_{x}$ in the absence of the *Policy Bundles* policies and $F{\left({d}_{x}\right)}_{p}$ is defined analogously. In words, this means that ${\Delta }_{x}$ measures the reduction in the area above the $F{\left({d}_{x}\right)}_{o}$ curve that results from *p* (see Fig. 2). Efficiency quantifies ${\Delta }_{all}$ and ${\Delta }_{low}$ per unit of cost required to implement *p*. It is defined in terms of two metrics ${\text{BCR}}_{all}$ and ${\text{BCR}}_{low}$, where:

3 $$\begin{array}{c}BC{R}_{x}={\Delta }_{x} /{C}_{p}\end{array}$$

and ${C}_{p}$ is the total cost of *p*.

${BCR}_{all}$ and ${BCR}_{low}$ can be considered as proxies for a conventional benefit–cost ratio (*BCR*); in this case, the optimal *Policy Bundle* to be selected is the one that maximises both ${\text{BCR}}_{all}$ and ${\text{BCR}}_{low}$. If no bundle satisfies this condition (i.e., one bundle maximises ${\text{BCR}}_{all}$ and another maximises ${\text{BCR}}_{low}$), the best bundle is selected based on stakeholder preferences for maximising ${\text{BCR}}_{all}$ versus ${\text{BCR}}_{low}$, which can be obtained using some sort of elicitation procedure (e.g., the analytical hierarchy process; Saaty 1987).

## Case study demonstration

We use the adapted framework to assess geophysics-informed, income-based, and spatially independent policies for Tomorrowville (Menteşe et al. 2023). Tomorrowville is a 5 km^2^ virtual urban testbed based on pertinent socio-demographic and geological features of Nairobi (Kenya) and Kathmandu (Nepal), designed to reflect a typical city in the Global South. It is underpinned by a geospatial database that contains detailed information on the testbed’s land uses, buildings, households, and individuals. The virtual domain consists of one shallow and one deep sedimentary basin, surrounded by basement rocks (Agrawal and McCloskey 2024). The development of Tomorrowville was coordinated and executed by an interdisciplinary team of researchers (e.g., urban planners, engineers, physical scientists, social scientists), leveraging geographically relevant literature and their respective expert judgment (Filippi et al. 2023). For instance, engineering expertise was used to determine the various building typologies of the buildings layer, whereas the spatial location of households and their association with different residential building types was defined by social scientists/urban planners based on distinct household income ranges and relevant urbanization patterns in the Global South (without any explicit consideration of geophysical information). We make use of Tomorrowville’s current urban layout, referred to as TV0 in Menteşe et al. (2023).

### Urban planning

**Fig. 3:**
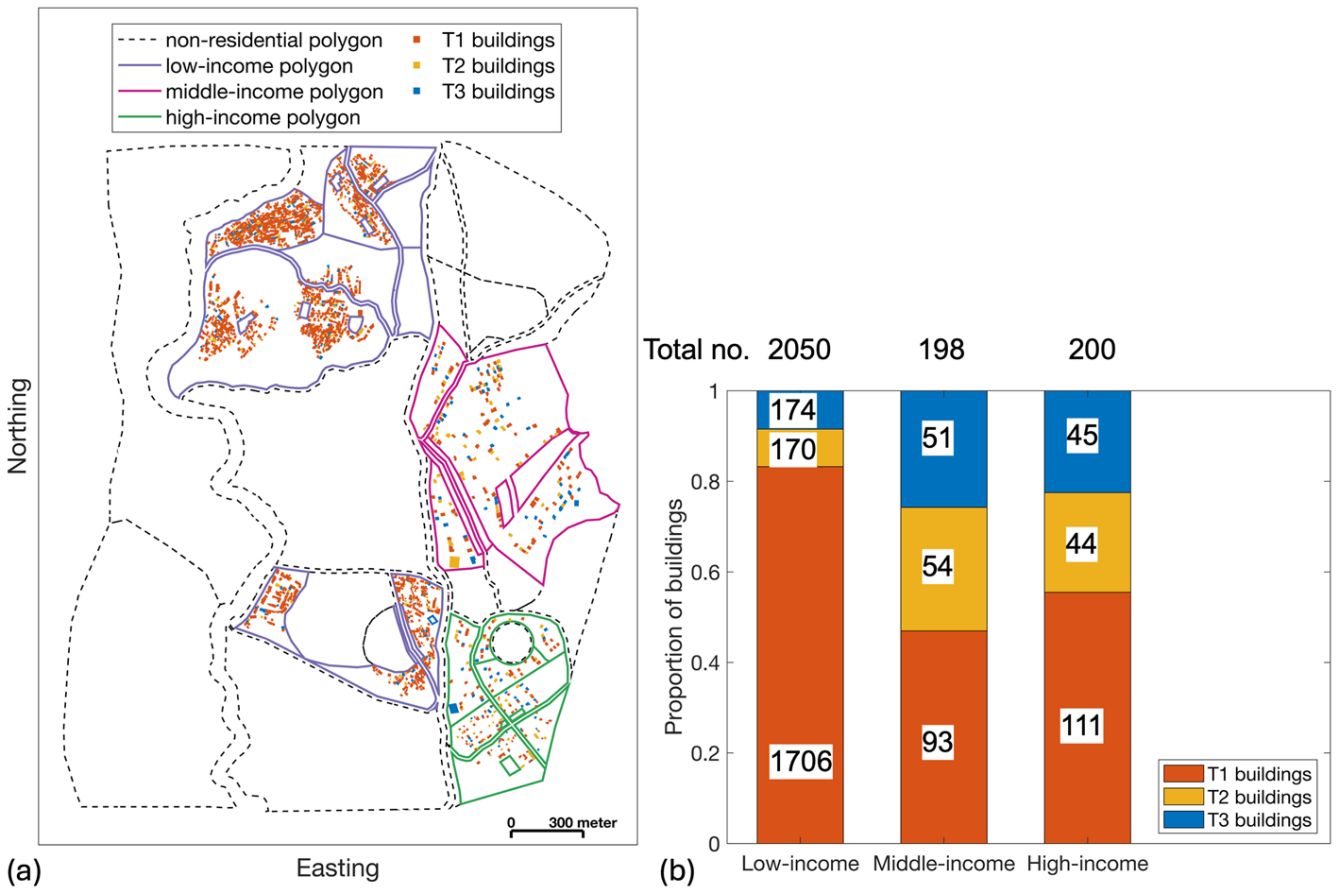
**a** TV0_pga_ buildings, including information on their building typology and associated land-use polygon (categorised based on income or as “non-residential”). **b** The proportion of different building typologies that feature across different income zones, which are defined based on the income level of the corresponding land-use polygons

In this application, the urban planning module contains the subset of TV0 incorporating buildings for which the physical vulnerability is characterised by PGA-based fragility relationships (herein referred to as TV0_pga_; details to follow in Sects. 3.3 and 3.4). TV0_pga_ comprises 2,441 residential buildings (with associated households/individuals) and seven critical facilities (either schools or hospitals); see Fig. 3a. 2,050, 198, and 200 of these buildings are located within low-income, middle-income, and high-income land-use polygons respectively. Building typologies within TV0_pga_ are classed as either “brick and mud walls” (T1), “brick and cement walls with flexible floor slabs” (T2), or “brick and cement walls with rigid floor slabs” (T3; see Gentile et al. 2022a for further information); the distribution of each typology by income level of the corresponding land-use polygon is provided in Fig. 3b.

#### Geophysical information

**Fig. 4:**
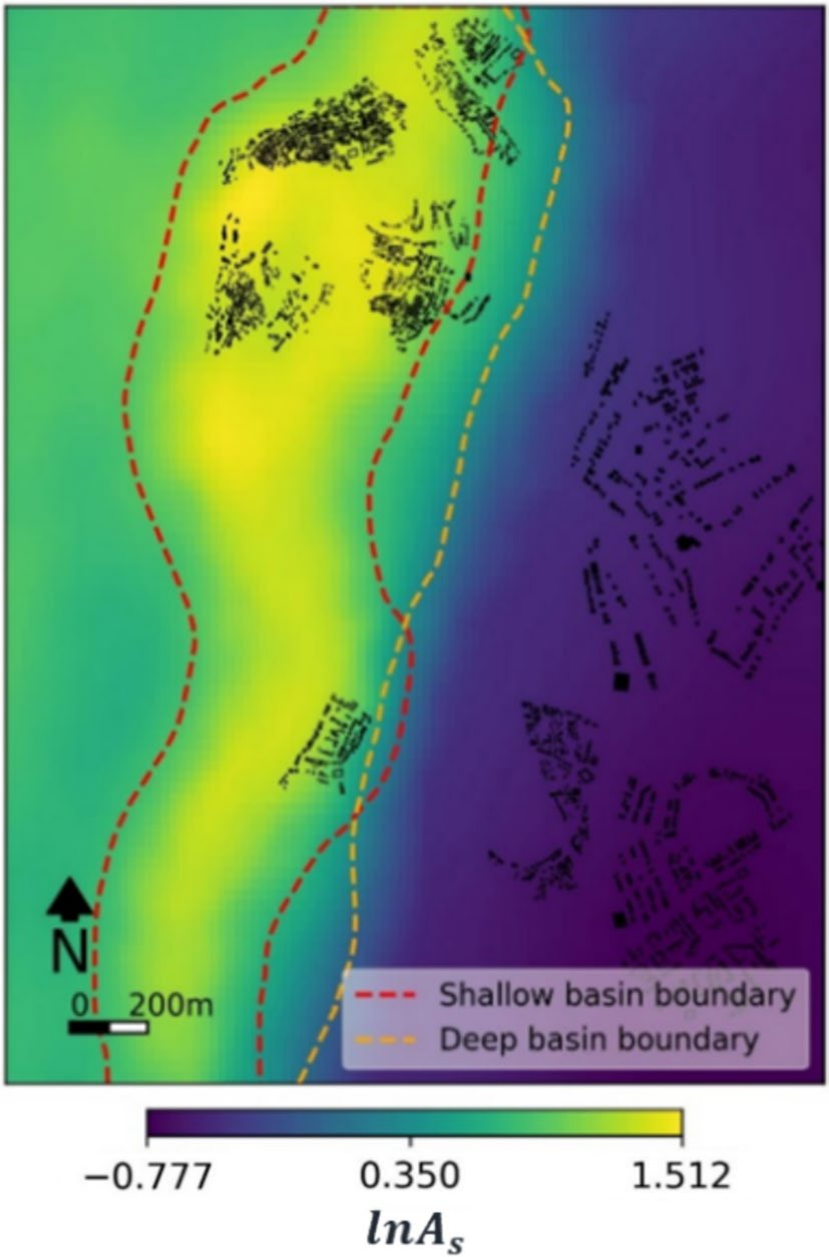
Spatial distribution of $ln{A}_{s}$ values across Tomorrowville. Also shown is the TV0_pga_ building portfolio (black polygons). This figure is adapted from Agrawal and McCloskey (2024)

The geophysical information used in the case study is centred around parameters describing local seismic site response, obtained from Agrawal and McCloskey (2024). For a site $s$, the (frequency-independent) seismic site response (or amplification) ${A}_{s}$ is calculated as:

4 $$\begin{array}{*{20}c} {A_{s} = \left( {\prod\limits_{{e \in N_{e} }} {u_{es} /u_{r} } } \right)^{{1/N_{e} }} } \\ \end{array}$$

where ${N}_{e}$ denotes a set of known earthquake scenarios and ${u}_{es}$ represents the site-specific ground-shaking intensity associated with the *e*th earthquake that occurs at an epicentral distance *r*. ${u}_{r}$ represents the mean ground-shaking intensity estimated for *r* and the magnitude of the *e*th earthquake, obtained using least-squares regression with data from across the set of ${N}_{e}$ earthquakes. More details can be found in Agrawal and McCloskey (2024). The ${N}_{e}$ earthquakes used to quantify ${A}_{s}$ are described in Sect. 3.3. Figure 4 maps ${A}_{s}$ values (in the logarithmic domain) across Tomorrowville. It can be seen that the basin area is associated with the largest ${A}_{s}$ values (highest amplification), due to wave trapping and resonance effects.

**Fig. 5:**
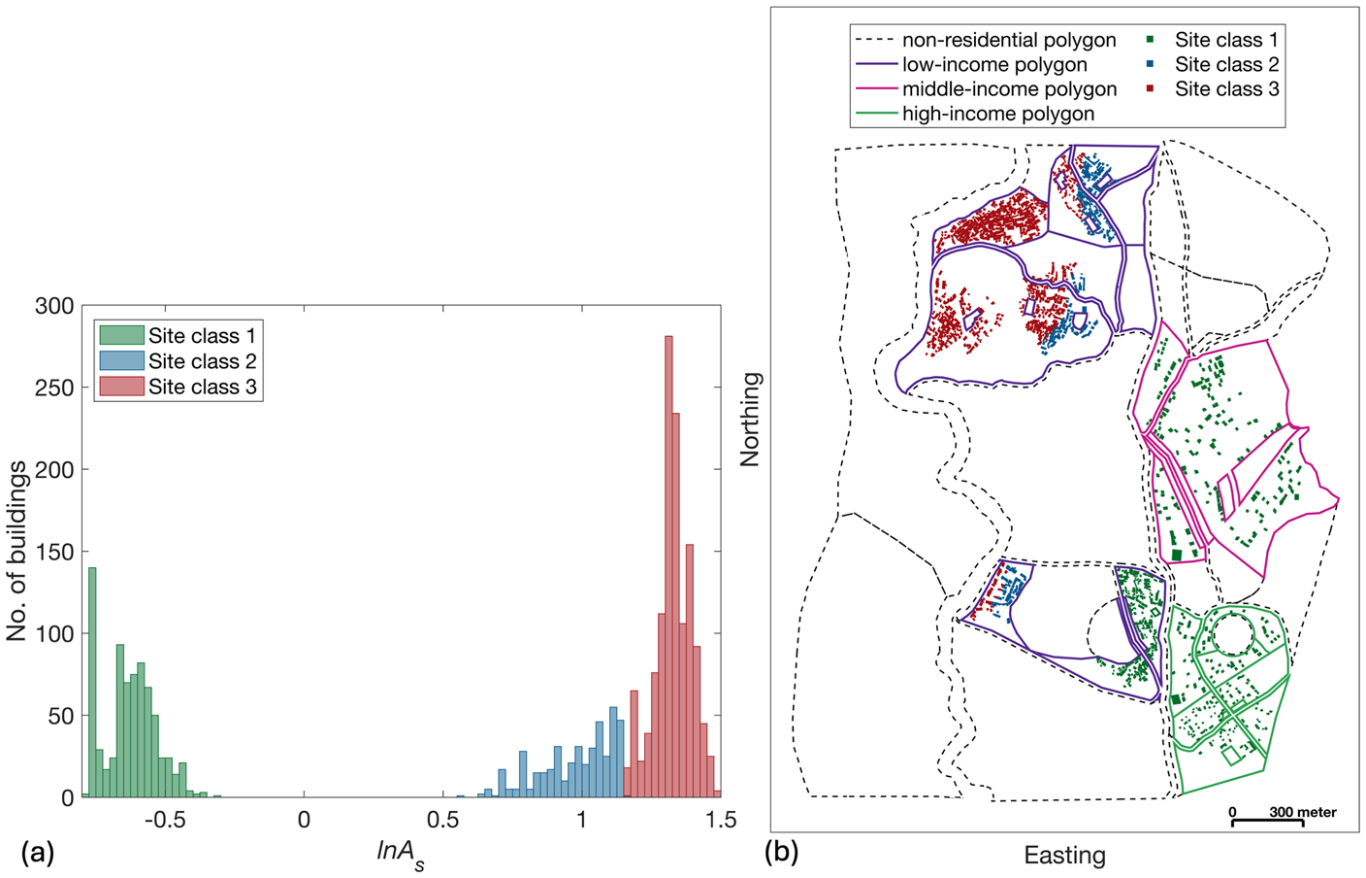
**a** Histogram of $ln{A}_{s}$ values at the locations of buildings in TV0_pga_, categorised in three site classes. **b** TV0_pga_ buildings colour-coded based on the corresponding site class

To facilitate the geophysics-informed policy design process (Sect. 3.2), we obtain $ln{A}_{s}$ value corresponding to each building location and group similar values together in a finite number of so-called site classes, using K-means clustering (Hartigan and Wong 1979). After some exploratory analysis, we settle on K = 3 (i.e., three site classes), which leads to statistically distinguishable clusters (*p*-value < 0.05) as confirmed by Analysis of Variance (Fisher 1921). The three site classes, numbered in order of increasing amplification, are shown in Fig. 5. Buildings in middle- and high-income zones (land-use polygons) are located exclusively within site class 1. Buildings in low-income zones are located within all three site classes; 62%, 21%, and 17% of these buildings are situated within site classes 1, 2 and 3, respectively. This configuration of buildings suggests that the lowest income populations of Tomorrowville are generally exposed to the highest ground-motion intensities.

### Policy bundles

We develop and assess five corrective DRM policies, which are treated as separate bundles. Each policy involves seismically retrofitting a subset of TV0_pga_ buildings that are selected on the basis of building typology, possibly in combination with information on income level of the associated land-use polygon or site class (see Table 1). We implement a low-cost retrofitting approach that involves placing Polypropylene (PP-band) mesh around outer walls, which is commonly used to upgrade low strength masonry buildings in countries of the Global South (Shrestha et al., 2012; Heydariha et al., 2019; Hoyos and Silva, 2022).

**Table 1:** Summary of policies (policy bundles) considered in this case study

Policy Number ($p$)	Building Typology Retrofitted	Income Level Targeted	Site Class Targeted	Number of Buildings Retrofitted (${N}_{Tx,p}$)		
				T1	T2	T3
1	T1	All	All	1910	0	0
2	T1	Low	All	1706	0	0
3	T1	All	3	1055	0	0
4 (3a)	T1 and T2	All	3	1055	108	0
5 (3b)	T1	All	2 and 3	1418	0	0

Policy 1 represents a spatially independent (uniform) approach to retrofitting that targets the most physically vulnerable building typology (see Sect. 3.4). Policy 2 is income-based, whereas Policies 3 to 5 can be described as geophysics-informed. Policies 4 and 5 can be regarded as more expensive variations of Policy 3 (targeting more site classes or building typologies in order of increasing concern), enabling us to evaluate the marginal efficiency advantages/disadvantages of possibly allocating more funding to the retrofitting process. Policies 1 to 5 are designed to realistically reflect real-world (pro-poor) policy making on earthquake risk management. Policy 1 represents a typical retrofitting policy that targets specific types of construction vulnerable to earthquakes (Gentile et al. 2022b). Policy 2 mirrors the Mexican government-led residential housing reconstruction program in the aftermath of the 2017 Mexico earthquake, which prioritised low-income families (Valle, 2024), whereas Policies 3, 4, and 5 broadly reflect the microzonation approach adopted by the Italian Government after the 2016–2017 seismic sequence in Central Italy, which accounted for local geophysical conditions in providing site-dependent requirements for retrofitting and reconstruction interventions (Moscatelli et al. 2020). Retrofitting alters the parameters of the fragility relationships used for affected buildings, as described in Sect. 3.4. ${C}_{p}$ is quantified as the sum of the relative cost of buildings to be retrofitted under each policy, given as:

5 $$\begin{array}{c}{C}_{p}={N}_{T1,p}+1.17\times 1.53\times {N}_{T2,p}\end{array}$$

where ${N}_{T1,p}$ and ${N}_{T2,p}$ are the number of T1 and T2 buildings to be retrofitted under *p*, respectively, 1.17 represents the relative construction cost of T2 buildings (140 EUR/m^2^) compared to T1 buildings (120 EUR/m^2^), and 1.53 represents the mean floor areas of T2 buildings (122.4 m^2^) compared to T1 buildings (80.2 m^2^). Construction cost and mean floor area data are obtained from Wang et al. (2023).

### Seismic hazard modelling

**Fig. 6:**
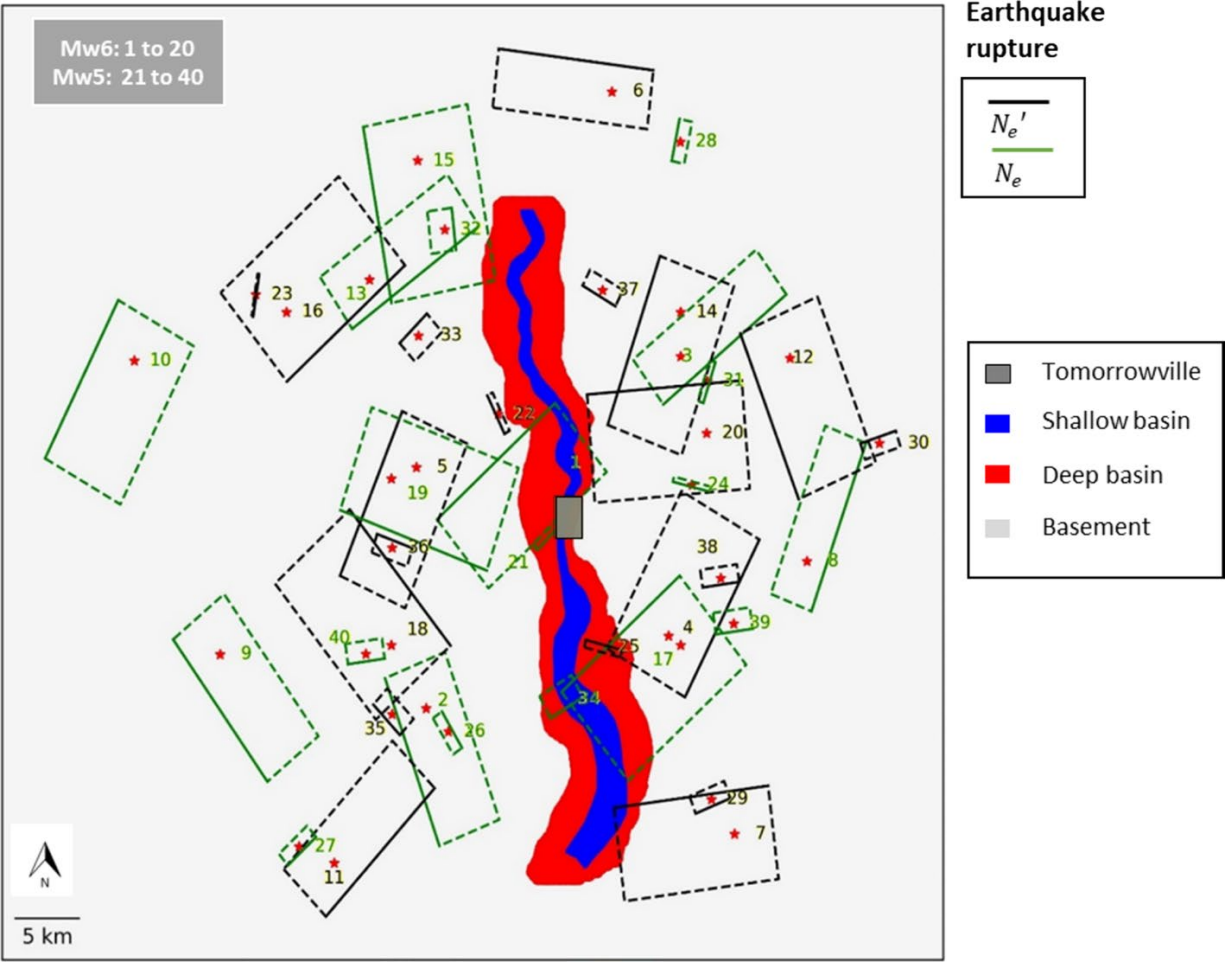
Map of the 40 thrust-faulting rupture scenarios considered in this study. Red stars denote hypocentres

We use a fully deterministic scenario-based approach to seismic hazard modelling in this study, based on the dominant tectonics of the domain that result in thrust-faulting earthquakes with magnitudes ${M}_{w}5.0$ and ${M}_{w}6.0$ (see Agrawal and McCloskey 2024). The scenario-based approach is adopted to ensure effective communication of risk to-and facilitation of associated decision making by-policymakers, who may not have a strong intuitive understanding of probability (Bonstrom et al. 2012). Twenty randomly located ${M}_{w}5.0$ and 20 ${M}_{w}6.0$ thrust-faulting earthquake ruptures (and their corresponding PGA values) are considered, which were simulated in Agrawal and McCloskey (2024) using the SPEED physics-based solver (Mazzieri et al. 2013). The 40 scenarios are divided into two sets of 20 (denoted as ${N}_{e}$ and ${{N}_{e}}^{\prime}$), each containing 10 ${M}_{w}5.0$ and 10 ${M}_{w}6.0$ events that are reasonably uniformly distributed in terms of both epicentral distance (to Tomorrowville) and hypocentral azimuth (see Fig. 6). Set ${N}_{e}$ is used to quantify ${A}_{s}$ in Eq. 4. Set ${{N}_{e}}^{\prime}$ provide twenty PGA ground-motion-field outputs of the *Seismic Hazard Modelling* module.

### Physical infrastructure impact

**Fig. 7:**
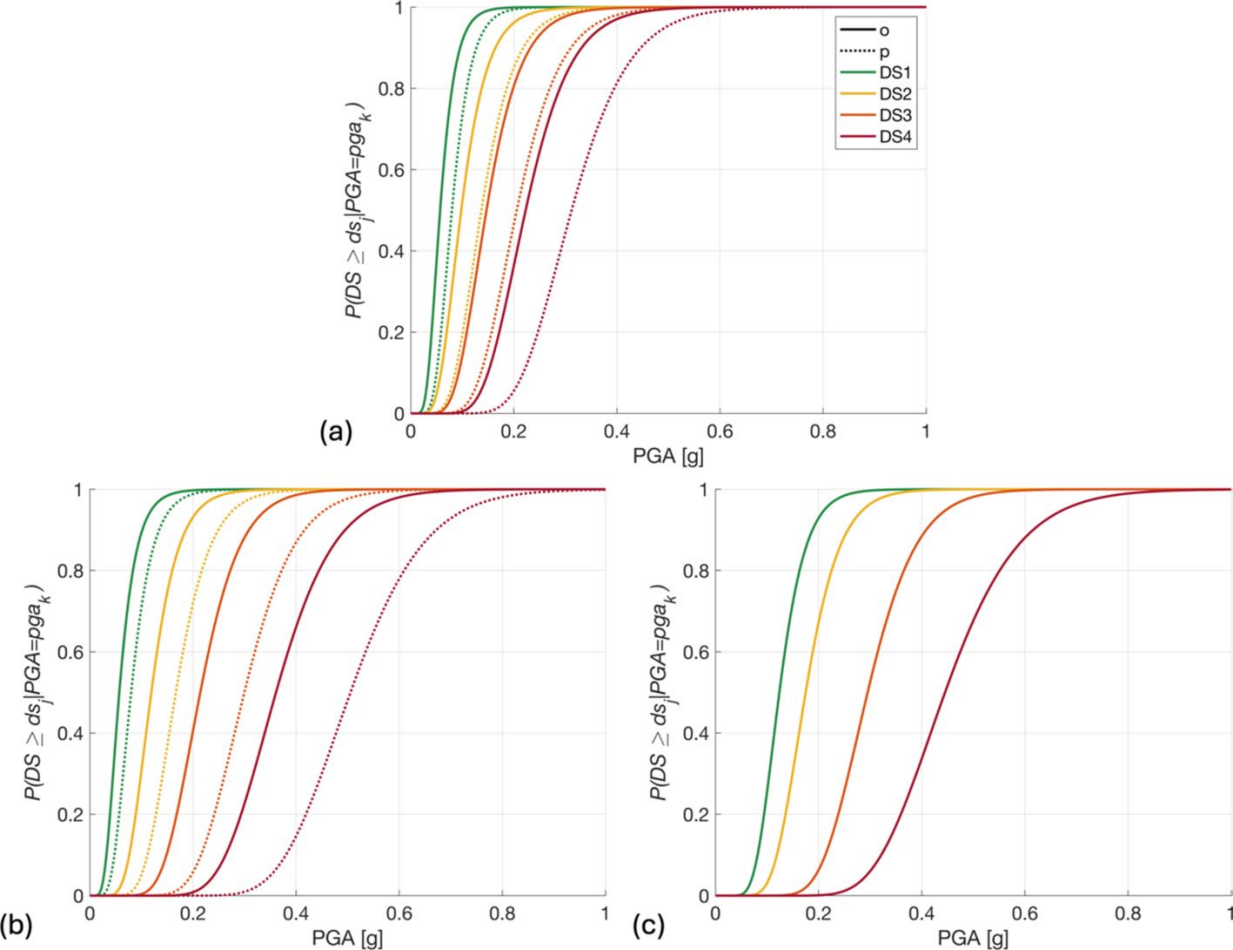
Fragility functions for **a** T1, **b**, T2, and **c** T3 buildings, in the non-retrofitted (*o*) and retrofitted (*p*) cases that feature across the various policies of Sect. 3.2

Fragility relationships for the unretrofitted TV0_pga_ buildings are obtained from Gentile et al., (2022a), which consider four damage states ($d{s}_{j}$): 1 (slight), 2 (moderate), 3 (extensive), and 4 (complete). For retrofitted buildings, the median parameters of these relationships are increased by 40% and the dispersions are decreased by 10%. These modifications are in line with the effects of using PP-band to retrofit a relatively similar building typology, i.e., one-storey clay brick unreinforced masonry, as detailed in Hoyos and Silva (2022); see Fig. 7. ${d}_{i}$ is defined as the average normalised damage state obtained across all ${{N}_{e}}^{\prime}$ events, i.e.,

6 $$\begin{array}{c}{d}_{i}=\frac{\overline{d{s }_{i}}}{\text{max}(\stackrel{-}{ds)} }= \left(\frac{1}{{{N}_{e}}^{{\prime}}}\right)\frac{\sum_{e\in {{{N}_{e}}}^{{\prime}} }\sum_{j=1}^{4}d{s}_{j}\times p\left(d{s}_{j} \right| {u}_{ei}) }{\text{max}(\stackrel{-}{ds)}}\end{array}$$

where ${u}_{ei}$ is the PGA value at the *i*^th^ building for the *e*th earthquake and $\text{max}(\stackrel{-}{ds)}$ is the maximum value of $\overline{d{s }_{i}}$ across all unretrofitted buildings and ${{N}_{e}}^{\prime}$ events. Therefore, ${d}_{i}$ ranges between 0 and 1.

**Fig. 8:**
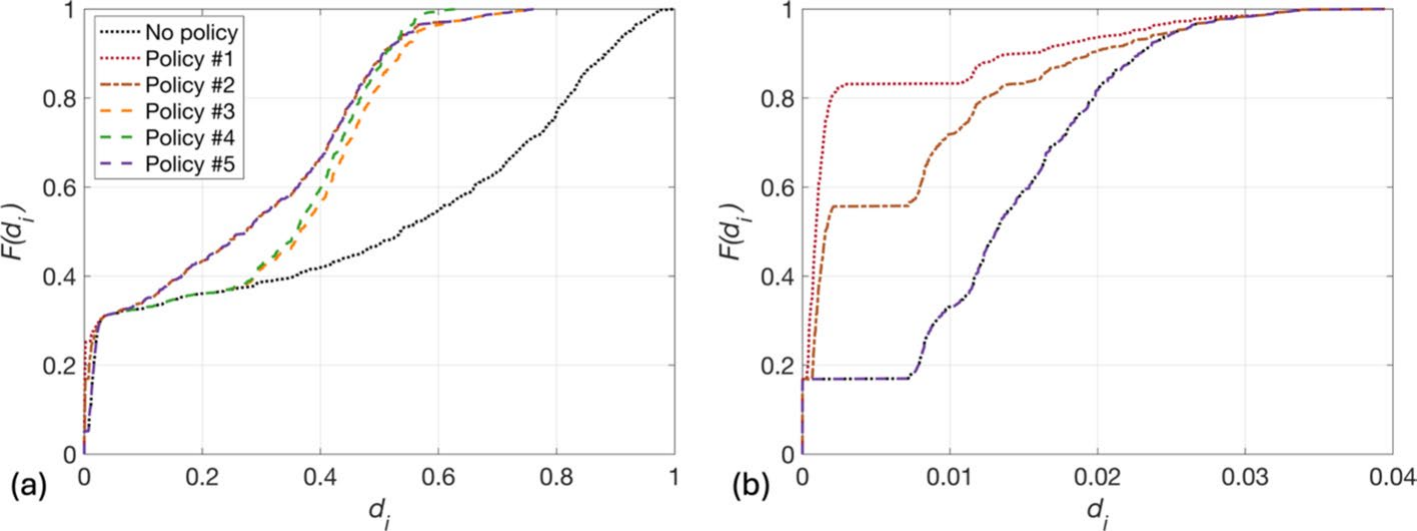
$F\left({d}_{i}\right)$ curves associated with each policy (and when no policy is implemented) for TV0_pga_ buildings in **a** all site classes and **b** site class 1

Figure 8 displays the performance of each policy in terms of $F\left({d}_{i}\right)$ values. Figure 8a indicates that policies 1, 2 and 5 result in almost identical distributions of ${d}_{i}$; differences between these policies only exist in the $F\left({d}_{i}\right)$ curves associated with site class 1 (see Fig. 8b). Policies 3 and 4, which target only site class 3, generally have higher ${d}_{i}$ values than policies 1, 2, and 5. Policy 4 produces slightly lower ${d}_{i}$ values than Policy 3, because it involves retrofitting more buildings.

### Social impact

**Fig. 9:**
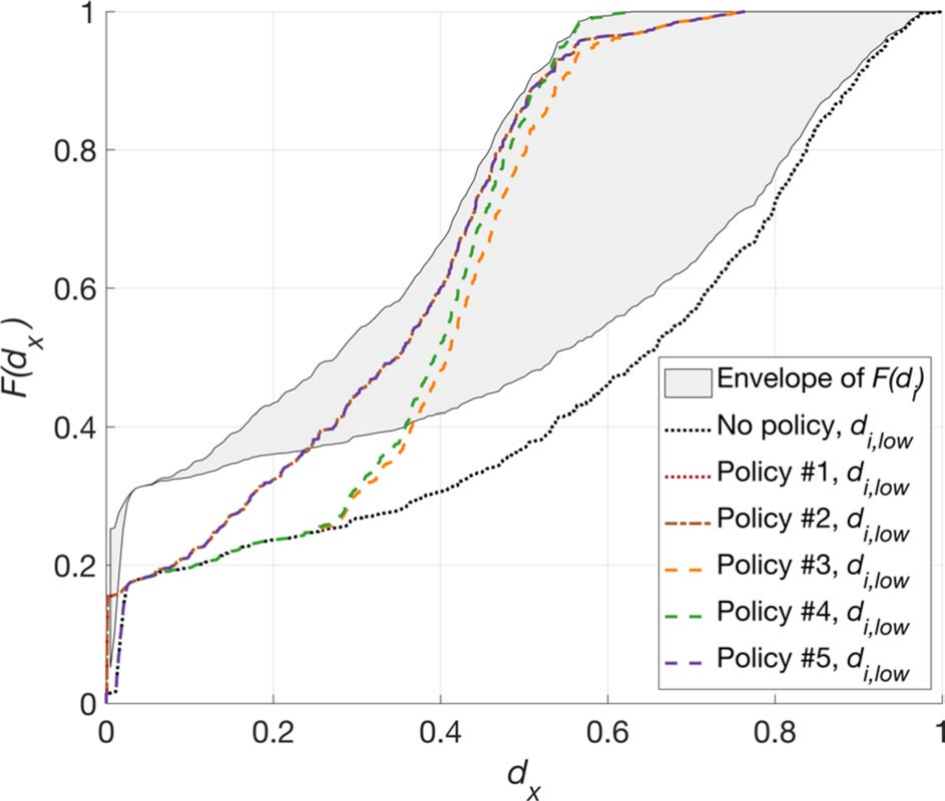
$F\left({d}_{i,low}\right)$ curves for each policy (and when no policy is implemented), compared to the boundary (envelope) of ${F(d}_{i})$ curves across different policies and when no policy is implemented

${d}_{i,low}$ values are defined as the subset of ${d}_{i}$ that pertain to TV0_pga_ buildings in low-income land-use polygons. Figure 9 depicts the reduction in ${d}_{i,low}$ values that result from each policy implementation. The $F\left({d}_{i,low}\right)$ curves are generally shifted to the right of the envelope of ${F(d}_{i})$ curves (which includes the case of no policy being implemented), indicating that, overall, low-income zones experience higher impact compared to all locations collectively. However, discrepancies between the mean ${d}_{i}$ and ${d}_{i,low}$ values are reduced across all considered policies (compared to the case of no policy implementation). These reductions are 39.0%, 40.2%, 31.7%, 38.1%, and 39.8% for Policies 1 to 5, respectively. Thus, the lowest difference between the mean ${d}_{i}$ and ${d}_{i,low}$ values is obtained for Policy 2, which involves retrofitting only T1 buildings falling exclusively within low-income polygons.

### Computed impact metrics

**Fig. 10:**
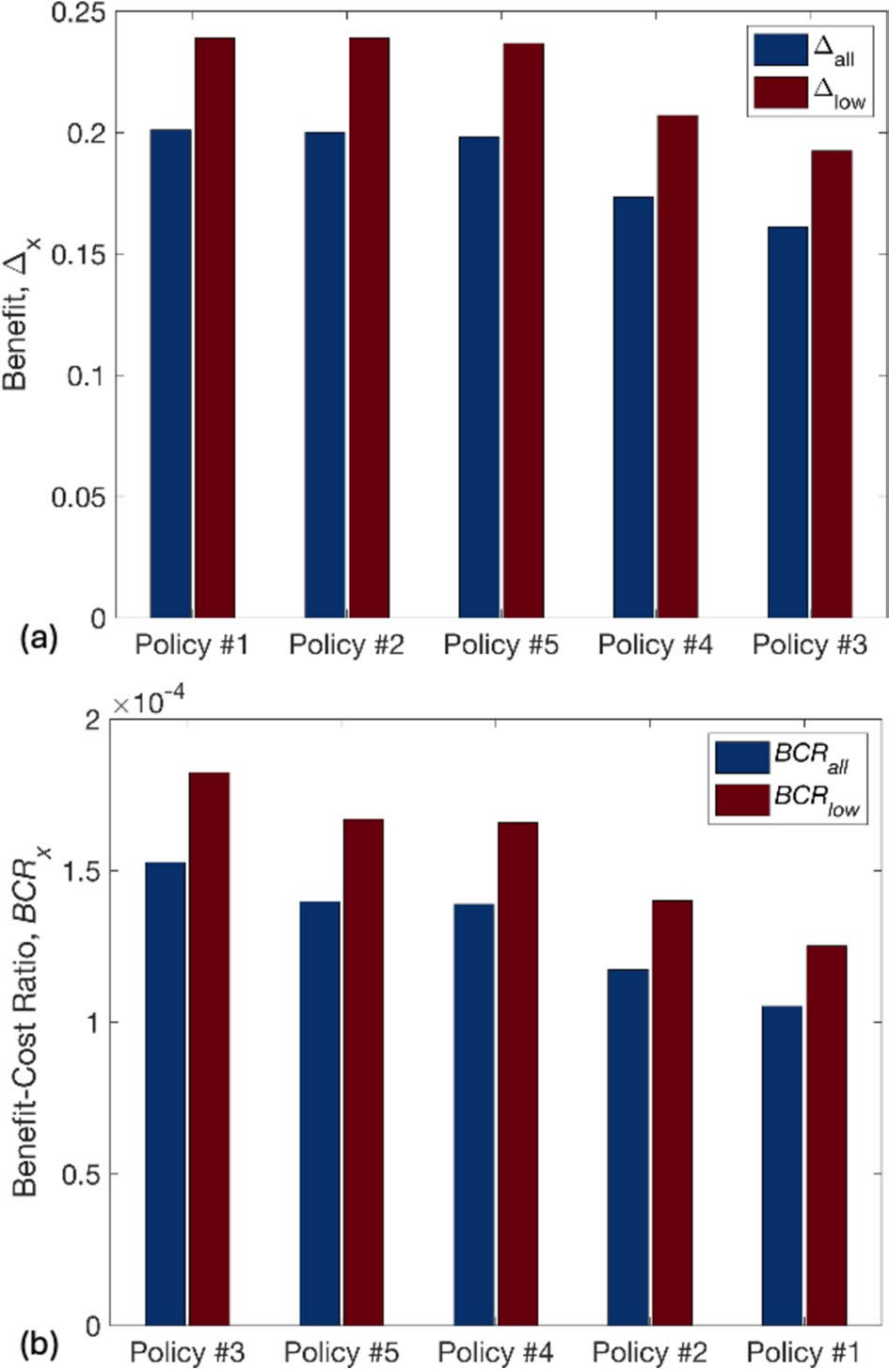
The **a** effectiveness and **b** efficiency of implementing each considered policy. Note that policies are organised in descending order of the corresponding metric values

Figure 10 plots ${\Delta }_{all}$, ${\Delta }_{low}$, $BC{R}_{all}$, and $BC{R}_{low}$ values associated with each policy. As expected, the trend in the effectiveness of policies (expressed in terms of both ${\Delta }_{all}$ and ${\Delta }_{low}$) reflects the number of buildings retrofitted, i.e., the most (Policy 1, joint with Policy 2 in the case of ${\Delta }_{low}$) and least (Policy 3) effective policies involve retrofitting the highest and lowest number of buildings, respectively. However, when the cost of policy implementation ${C}_{p}$ is factored in through $BC{R}_{all}$ and $BC{R}_{low}$, the geophysics-informed policies (Policy 3, followed by Policies 5 and 4) are deemed the most efficient according to both metrics. Thus, the final output of the framework in this case is a *Visioning Scenario* consisting of TV0_pga_ with retrofitted T1 buildings in site class 3.

This finding suggests a few interesting insights: (1) When limited budget is available, geophysical information can help with strategising a cost-efficient disbursement of resources for earthquake DRM; and (2) geophysics-informed policies can be more pro-poor from an efficiency (cost–benefit) perspective than policies that are specifically designed to target low-income groups, indicating that geophysical information has the potential to enhance equity in DRM decision-making without the need to explicitly consider possibly sensitive income cut-off thresholds.

## Conclusions

This study proposes a framework for assessing both the effectiveness and the efficiency (benefit per cost) of geophysics-informed DRM policies in reducing earthquake impact (risk) across different socio-economic groupings. The performance of these policies is benchmarked against those that are explicitly income-based or more spatially independent. The framework is based on the Tomorrow’s Cities Decision Support Environment for pro-poor risk-sensitive urban planning (Cremen et al. 2023; Galasso et al. 2021) and resulting approaches for designing relevant policies (Wang et al. 2023).

We showcased the framework by designing and assessing various earthquake DRM policies for Tomorrowville, a virtual urban testbed designed with interdisciplinary expertise to reflect a typical Global South setting. These policies involved seismically retrofitting various buildings that were selected based on either: (1) defined site classes that account for seismic amplification (geophysics-informed); (2) the income levels of the associated population; or (3) their physical vulnerability (in a spatially independent sense). We find that while the most effective policy is the spatially independent one that retrofits the most buildings (as expected), the most efficient policies are those that strategically target a subset of these buildings based on high-resolution geophysical information. This finding remains the same, regardless of whether efficiency is examined across all or just low-income populations. To summarise, the case study application suggests that geophysics-informed policies hold potential for providing an economically viable approach to pro-poor DRM, without the need to consider possibly controversial income-based implementation cut-off thresholds.

Geophysics-informed policies are pro-poor for Tomorrowville, because of the significant spatial correlation that exists there between income and site class. Low-income communities predominantly reside in areas with the highest seismic amplification (i.e., the sedimentary basin along and close to the riverbank, comprised of loosely consolidated sediment fill), whereas higher income groups are located in the more stable geological basement. This trend mirrors real-world contexts, in which the dynamics of land and housing markets frequently force the poorest in society to live in the most seismically hazardous areas, such as low-lying floodplains near rivers (Nakagawa et al. 2009; Boelhouwer et al., 2018; Shi and Naylor, 2023; Bangalore et al. 2019; Kawasaki et al. 2020). Our work fills a crucial gap in the literature by explicitly highlighting and providing a better understanding of the consequences associated with the close relationship between geophysical characteristics of the ground and the overlying social earthquake risk circumstances of urban systems (within the context of residential buildings, schools, and hospitals). In conclusion, the framework can be used to facilitate decision making on equitable DRM that is informed and supported by defensible and objective geoscientific information.

## Data Availability

The data and code for implementing the case study on Tomorrowville are made available through the following repository: https://github.com/himansh78/DRM_policy_assess. Original data related to Tomorrowville can be found here: https://github.com/TomorrowsCities/Tomorrowville.
